# Esophageal Dysmotility in Chronic Hemodialysis Patients After Ingestion of Liquids With Different Viscosities

**DOI:** 10.4021/gr300w

**Published:** 2011-03-20

**Authors:** Clovis Massato Kuwahara, Lucilene Rosa-e-Silva, Altair Jacob Mocelin, Miriam Zebian, Rose Meire Albuquerque Pontes, Roberto Oliveira Dantas

**Affiliations:** a Department of Internal Medicine, Londrina State University, Health Center, Londrina, State of Parana, Brazil; b Department of Internal Medicine, Ribeirao Preto School of Medicine, University of Sao Paulo, Ribeirao Preto, State of Sao Paulo, Brazil

**Keywords:** Chronic renal failure, Esophageal manometry, Esophageal motility, Hemodialysis, High viscous substance

## Abstract

**Background:**

Previous studies assessing esophageal motility in chronic renal failure (CRF) patients had no consistency in their findings. These studies evaluated esophageal contractility in response to dry/water swallows. Our aim was to reassess esophageal motility in CRF patients to better define its abnormalities. To unmask minor defects not seen in conventional dry/water manometry we also evaluated esophageal contractility in response to a highly viscous substance.

**Methods:**

Fifteen controls and nine asymptomatic CRF patients underwent esophageal manometry with dry swallows, swallows of 5 mL of water (1 centipoise) and 5 mL of sugar cane syrup (24500 centipoise). CRF patients were compared with controls for esophageal motility parameters, considering each type of swallow (dry/water/syrup).

**Results:**

CRF patients had: tendency for higher lower esophageal sphincter (LES) resting pressure (P = 0.09); shorter LES relaxation duration after dry/water/syrup swallows (P = 0.0001, P < 0.0001, P = 0.0001, respectively); higher amplitude of proximal contractions after dry/water/syrup swallows (P = 0.008, P = 0.01, P = 0.04); tendency for longer duration of distal contractions after dry/water/syrup swallows (P = 0.07, P = 0.04, P = 0.09); lower velocity of distal contractions after dry/water/syrup swallows (P = 0.006, P = 0.09, P = 0.02); and higher incidence of multi-peaked contractions after dry/water/syrup swallows (P = 0.03, P = 0.0001, P < 0.0001).

**Conclusions:**

Esophageal motility dysfunction can be a sub-clinical manifestation in CRF patients. Data also showed that swallows of a highly viscous liquid did not help to detect minor esophageal dysmotility in these patients.

## Introduction

Gastrointestinal (GI) symptoms are frequently present in patients with chronic renal failure (CRF) [1]. Some of these symptoms such as nausea, vomiting, heartburn, and dysphagia may be related to esophageal dysmotility. However, few studies have evaluated the esophageal motor function in CRF patients. Symptomatic patients were described as having esophageal motility patterns of diffuse spasm or achalasia [2] yet there was no consistency in the abnormal findings for asymptomatic patients [3, 4].

Previous studies used manometry to evaluate the esophageal contractility in response to dry and/or water swallows [2-4]. A highly viscous substance has been employed in esophageal manometry [5-7] in order to stress control mechanisms of esophageal motility, thereby unmasking minor defects not revealed by dry and water swallows. However there are no studies evaluating esophageal motor function in CRF patients using a highly viscous substance.

Our aim was to reassess the sub-clinical esophageal contractility in CRF patients receiving hemodialysis treatment to better define which abnormalities are present in these patients. Also, by using a highly viscous substance we hoped to find esophageal motor abnormalities not otherwise revealed by conventional manometry.

## Materials and Methods

This protocol was approved by the local Ethics Committee with informed, written consent obtained from each subject.

Nine patients with CRF were studied (all male; median age: 46 years, range: 25 - 60 years). All of them had been undergoing hemodialysis three times a week. The clinical characteristics of the patients are shown in Table 1. None of the patients had a clinical diagnosis of systemic disease affecting GI motility such as neurologic diseases, muscular diseases, amyloidosis or collagen vascular disorders. All patients had normal levels of blood glucose and a negative serological test for Chagas disease.

**Table 1:** Clinical Characteristics of Chronic Renal Failure Patients Receiving Hemodialysis Treatment

Patient	Primary renal disease	Dialysis duration	Urea*	Creatinine*	Calcium*	Phosphorus*	Medication
		(months)	(mg/dL)^a^	(mg/dL)^b^	(mg/dL)^c^	(mg/dL)^d^	
1	CGN	168	120	11.9	8.3	5.5	A
2	CGN	180	130	13.6	8.0	5.9	A
3	PKD	96	195	17.1	8.3	6.1	A
4	CGN	108	152	14.2	9.2	5.2	A
5	CGN	204	170	15.3	8.8	5.3	A
6	CGN	72	102	9.8	8.0	5.1	A
7	PKD	58	120	11.9	8.1	5.4	A
8	CGN	144	94	9.1	8.5	5.0	A
9	CGN	21	153	13.5	8.3	4.8	A

CGN: chronic glomerulonephritis; PKD: polycystic kidney disease; A: vitamin preparations; normal ranges: (a) 10 - 25 mg/dL, (b) 0.7 - 1.4 mg/dL, (c) 8.5 - 10.5 mg/dL, (d) 3.0 - 4.5 mg/dL. *values obtained the day the patients underwent the manometry examination.

The control group consisted of 15 healthy volunteers without any esophageal symptoms (8 males, 7 females; median age: 40 years, range: 26 - 60 years). There was no statistical difference in age between CRF patients and controls (U = 61; P = 0.46).

Neither the patients nor the controls were chronic alcoholics. None of them had previous surgery in the GI tract nor were taking drugs that directly affect the GI motor function.

All subjects had a clinical interview and were asked about the presence of the following symptoms: heartburn, regurgitation, dysphagia, nausea, vomiting, globus, odynophagia and chest pain. All patients underwent an upper GI endoscopy.

For the esophageal motility assessment, patients and controls underwent an esophageal manometry study after an overnight fast. All patients had this study done one day after their hemodialysis session.

Serum urea, creatinine, calcium, and phosphorus levels were determined from a blood sample obtained from each patient on the day they underwent the manometry examination.

We used an esophageal manometry protocol as previously described [6, 7]. The studies were carried out with an 8 lumen water-perfused catheter measuring 4.5 mm in external diameter and 0.8 mm in internal diameter (Arndorfer Specialities Inc., Greendale, WI, USA). The four distal openings of the catheter were at the same level at 90° angles and were used to measure the lower esophageal sphincter (LES) pressure. The four proximal openings were spaced 5 cm apart at 90° angles to allow sampling of several sites in the esophageal body at the same time. The lumens of the catheter were connected to external pressure transducers which in turn were connected to a PC polygraph HR (Synetics Medical, Stockholm, Sweden). The measurements obtained by the polygraph were recorded on a computer.

The catheter was passed into the stomach through the nose, with the subject in the supine position.

The pressure of the LES was recorded using the 4 distal catheter lumen recordings and the rapid pull through technique [8]. The pressure was given by the mean of the values found in the 4 distal lumens. LES pressures were recorded in triplicate. The LES pressure for each patient was the mean of the three pressures recorded.

In order to study the esophageal body and the LES relaxation duration, the distal catheter opening was positioned at the level of the LES, and three other openings located at 5, 10, and 15 cm above the LES. With the catheter in this position, 30 swallows were recorded: 10 dry, 10 wet (5.0 mL of distilled water, 1 centipoise) and 10 of a highly viscous substance (5.0 mL of sugar cane syrup, 24500 centipoise). The order of swallows was random. After each swallow, contractile wave amplitude, duration, and velocity were determined at 5, 10 and 15 cm above the LES as described by Richter et al [9]. Also, LES relaxation duration was measured from the beginning of the descending pressure curve to the beginning of the ascending curve recorded by the distal catheter opening positioned at the LES. The results were given by the mean of the 10 measurements obtained for each type of swallow (dry, water and sugar cane syrup). The contractile wave amplitude, duration and velocity were determined by the mean of the 10 measurements obtained for each type of swallow, at the different sites (5, 10 and 15 cm above the LES).

In the esophageal body, we also noted the presence of failures of contraction (wave amplitude less than 10 mmHg or complete absence of motor activity), non-peristaltic contractions (with simultaneous onset in different recording sites), and multi-peaked contractions (with two or more peaks).

### Statistical analysis

For the numeric variables, we used the Mann-Whitney test to compare CRF patients and controls. LES resting pressure was compared between both groups. LES relaxation duration was compared between the groups for each type of swallow (dry, water and sugar cane syrup). Contractile wave amplitude, duration and velocity were compared between both groups at the different levels of the esophagus (5, 10 and 15 cm above LES) for each type of swallow. Median and range of the numeric variables are reported for both groups.

For the nominal variables (failed, non-peristaltic and multi-peaked contractions) we used the Fisher Exact test to compare CRF patients and controls for each type of swallow.

A P value < 0.05 was considered statistically significant.

## Results

None of the CRF patients had esophageal symptoms or abnormalities in their upper GI endoscopy examination. On the day the patients underwent the manometry evaluation, they had high serum urea and creatinine levels, normal serum calcium levels, and slightly high serum phosphorus levels (Table 1).

### Parameters of the lower esophageal sphincter (LES)

LES resting pressure tended to be higher in CRF patients than in controls but the difference did not achieve statistical significance (Table 2). LES relaxation duration was shorter in CRF patients than in controls for all types of swallows (Table 2).

**Table 2:** Lower Esophageal Sphincter (LES) Pressure and LES Relaxation Duration in Controls and in Patients With Chronic Renal Failure (CRF)

LES parameters	Type of swallow	Controls (n = 15)	CRF patients (n = 9)	P-values
LES resting pressure (mm Hg)		21.0 (14.0 - 49.0)	42.0 (7.0 - 95.0)	0.09^b^
	Dry	9.4 (6.6 - 10.8)	5.6 (3.4 - 8.3)	0.0001^a^
LES relaxation duration (seconds)	Wet	9.9 (8.5 - 12.6)	7.5 (5.7 - 8.4)	< 0.0001^a^
	Syrup	9.7 (6.6 - 11.8)	7.6 (4.3 - 8.0)	0.0001^a^

The results are shown as median (range).

a: P < 0.05, statistically significant

b: 0.05 < P < 0.1, tendency towards significance

### Numeric parameters of the contractile waves

Amplitude was higher in CRF patients than in controls in the proximal esophagus (15 cm above the LES) for all types of swallows (Table 3). Duration tended to be longer in CRF patients than in controls in the distal esophagus (5 cm above the LES) for all types of swallows but achieved a statistical significance only for water swallows (Table 3). Velocity tended to be lower in CRF patients than in controls in the distal esophagus (10 to 5 cm above the LES) for all type of swallows, achieving a statistical significance for dry and syrup swallows (Table 3).

**Table 3:** Amplitude, Duration and Velocity of Esophageal Contractions in Controls and in Patients With Chronic Renal Failure (CRF)

Contraction parameters	Type of swallow	Esophageal site	Controls (n = 15)	CRF patients (n = 9)	P-values
Amplitude (mm Hg)	Dry	15	31 (12 - 84)	38 (31 - 92)	0.008^a^
		10	46 (25 - 143)	46 (12 - 91)	0.59
		5	64 (28 - 187)	70 (24 - 111)	0.95
	Wet	15	33 (24 - 121)	96 (29 - 120)	0.01^a^
		10	64 (35 - 182)	130 (21 - 151)	0.19
		5	116 (50 - 313)	150 (23 - 235)	0.34
	Syrup	15	36 (20 - 89)	56 (24 - 105)	0.04^a^
		10	72 (30 - 170)	68 (20 - 125)	0.95
		5	103 (30 - 224)	81 (25 - 210)	0.90
Duration (s)	Dry	15	3.6 (2.3 - 5.2)	3.7 (1.1 - 5.3)	0.60
		10	3.9 (3.2 - 5.9)	4.5 (2.1 - 6.5)	0.19
		5	4.3 (2.8 - 8.8)	5.1 (4.2 - 7.3)	0.07^b^
	Wet	15	3.5 (2.5 - 5.2)	4.2 (1.9 - 5.2)	0.09^b^
		10	4.5 (2.7 - 5.7)	5.7 (1.8 - 7.1)	0.003^a^
		5	4.9 (3.4 - 12.7)	6.0 (4.5 - 10.1)	0.04^a^
	Syrup	15	3.9 (2.2 - 4.9)	3.9 (1.1 - 5.3)	1.00
		10	4.2 (3.4 - 5.9)	5.3 (2.0 - 6.6)	0.21
		5	4.7 (3.2 - 10.8)	5.3 (4.6 - 7.7)	0.09^b^
Velocity (cm/s)	Dry	15-10	3.9 (1.8 - 7.0)	3.7 (2.4 - 5.0)	0.81
		10-5	4.8 (1.9 - 8.2)	2.5 (1.6 - 6.4)	0.006^a^
	Wet	15-10	3.2 (1.5 - 5.4)	3.3 (1.8 - 4.5)	1.00
		10-5	4.0 (1.8 - 6.9)	2.5 (1.5 - 10.4)	0.09^b^
	Syrup	15-10	3.4 (1.8 - 7.0)	2.8 (2.0 - 4.4)	0.86
		10-5	4.3 (1.8 - 7.0)	2.5 (1.4 - 7.6)	0.02^a^

The results are shown as median (range).

a: P < 0.05, statistically significant

b: 0.05 < P < 0.1, tendency towards significance

### Nominal parameters of the contractile waves

#### Failure of contraction

There was no difference in failure of contraction incidence between CRF patients and controls for dry swallows (25% vs. 17%, P = 0.1) as well as for water swallows (4% vs. 4%, P = 1.0) and for syrup swallows (17% vs. 8%, P = 0.15).

#### Non-peristaltic contraction

There was no difference in non-peristaltic contraction incidence between CRF patients and controls for dry swallows (17% vs. 8%, P = 0.15) as well as for water swallows (4% vs. 4%, P = 1.0) and for syrup swallows (4% vs. 4%, P = 1.0).

#### Multi-peaked contraction

The incidence was higher in CRF patients than in controls for dry swallows (29% vs. 8%, P = 0.003) as well as for water swallows (33% vs. 4%, P = 0.0001) and for syrup swallows (38% vs. 8%, P < 0.0001).

## Discussion

The results of this study show that esophageal motor dysfunction can be a sub-clinical manifestation in CRF patients, which can occur at the LES as well as in the esophageal body. Our results also show that swallowing a highly viscous liquid does not help in the detection of minor esophageal motor defects in CRF patients.

Other similar studies also found that esophageal dysmotility is a sub-clinical manifestation of chronic uremia [3, 4]. However, only one of these studies [3] showed results similar to ours. At the esophageal body level, Siamopoulos et al [4] observed higher incidence of biphasic and triphasic esophageal contractions in CRF patients. Our study and Dogan et al [3] showed these results but also higher amplitude, longer duration, and lower velocity of the esophageal contractions. The difference in the results of Siamopoulos et al [4] may be explained by the different protocol employed in their study. They evaluated the esophageal motor function after 3 wet and 3 dry swallows while we and Dogan et al [3] studied this function after multiple swallows (10 and 6, respectively). Multiple swallows may minimize the variations in the esophageal body contractions producing more reliable results.

At the LES, Siamopoulos et al [4] did not observe differences between controls and CRF patients for resting pressure, while we and Dogan et al [3] observed a tendency for higher LES resting pressure, although the difference did not achieve statistical significance in both studies. It is possible that statistical significance would be achieved if a larger number of patients had been evaluated. We also observed that LES relaxation duration was shorter in CRF patients than controls. Siamopoulos et al [4] did not evaluate this while Dogan et al [3] did not observe differences between controls and patients. The shorter LES relaxation duration observed in our asymptomatic CRF patients is more compatible with the findings observed by Francos et al [2] in symptomatic CRF patients. They found high LES resting pressure in all five patients of their study as well as incomplete LES relaxation in two patients and no LES relaxation in another. We did not observe achalasia in our CRF patients, but it is unknown if the shorter LES relaxation duration in asymptomatic patients will develop to achalasia in symptomatic patients. Further studies with high resolution manometry may help to clarify this.

Our study has a small sample size for methodological reasons. We did not include CRF patients with systemic diseases that could affect GI motility such as amyloidosis, diabetes mellitus or collagen vascular disease. It is known that diabetic nephropathy is a major cause of CRF in patients undergoing hemodialysis [10, 11]. Also, we did not include in our study patients taking any medication that could interfere with GI motility. It is known that a large number of CRF patients need to take medications such as antihypertensive drugs that could interfere with the GI motility [10, 11]. Other studies evaluating esophageal contractility in CRF patients also had small sample sizes [2-4, 12]. However, we believe that, except for the LES resting pressure, the small sample size did not influence our results since the findings were very consistent.

Previous studies evaluated esophageal motor function in chronic uremia including patients of both genders [3, 4, 12]. Even though it was not our intention, we evaluated only male CRF patients. However, we believe that it has not affected our results since previous studies showed that gender had no effect on esophageal motility [13].

The clinical relevance of the motility data observed by our study and by Dogan et al [3] is uncertain. A variety of GI symptoms described in CRF patients [1] may be related to esophageal dysmotility, such as heartburn, dysphagia, nausea, and vomiting. Heartburn has been related to Gastroesophageal Reflux Disease (GERD) in these patients [14, 15], while dysphagia is shown to be related to achalasia and esophageal diffuse spasm [2]. None of the motility data observed at the esophageal body level in our patients has been reported as playing a role in GERD, achalasia or diffuse esophageal spasm. Regarding the multi-peaked esophageal contractions, some authors consider them to be normal variants and not abnormalities. However, higher incidence of byphasic and multi-peaked contractions were described in some esophageal motiltiy disorders such as diabetes mellitus and noncardicac chest pain [16-18]. Moreover, Francos et al [2] observed that three out of five symptomatic CRF patients had multi-peaked contractions.

The results we observed at the LES level (tendency for high LES resting pressure and shorter LES relaxation duration), have not been described as playing a role in GERD. Abnormalities in transient LES relaxation, a parameter not yet evaluated in CRF patients, may be involved in their GERD. More studies are necessary to clarify this issue.

Previous studies evaluated the esophageal motor function in CRF patients in response to dry and/or water swallows [3, 4]. We also evaluated the esophageal motor function in response to a highly viscous substance swallow. We employed the highly viscous substance in the manometry in order to stress control mechanisms of esophageal motility, hoping to unmask minor defects not revealed by dry and water swallows [5-7]. However, the data we observed in the esophageal motility of CRF patients were consistently observed after dry, water and sugar cane syrup swallows. Dooley et al [5] hypothesized that esophageal contractility is changed by modifications in bolus viscosity due to a reflex mediated through esophageal stretch receptors responding to the presence of an intra-luminal bolus. The fact that we did not find minor abnormalities in our CRF patients after sugar cane syrup swallows implies that the mechanisms involved in the results we found are not related to myo-neural modulation. Siamopoulos et al [4] reinforce this in their evaluation of cardiovascular autonomic dysfunction of CRF patients, which found that 11 out of 16 patients had autonomic neuropathy. However, the esophageal dysmotility observed in their patients was not related to the autonomic dysfunction.

Uremic myo-neuropathy is not the only cause of GI motility disturbance in CRF patients. Other factors such as changes in serum electrolyte (calcium, phosphorus) levels as well as peptide plasma levels [19-25] can play a role in the esophageal dysmotility observed in our patients. We believe that the serum electrolytes levels did not influence our results since the values obtained from our patients’ blood samples collected on the day they underwent manometry were normal or slightly out of the normal range (Table 1). Regarding the peptide plasma levels, a few studies showed that some peptides such as gastrin, motilin, neuropeptide Y, substance P, pancreatic polypeptide, and vasoactiveintestinal peptide are increased in CRF patients receiving hemodialysis treatment [19-27]. However, there are no studies evaluating the relationship of abnormal peptide plasma levels and esophageal dysmotility in these patients. Future studies need to evaluate this.

In conclusion, our data shows that CRF patients present a sub-clinical esophageal motor dysfunction, helping to clarify the controversy about the type of dysmotility observed in these patients. Our results also show that swallows of a highly viscous liquid does not help to detect minor esophageal motor dysfunction in asymptomatic CRF patients, which implies that myo-neural modulation was not involved in the mechanisms causing the data we observed.
